# A novel homozygous initiation codon variant associated with infantile alpha‐Bcrystallinopathy in a Chinese family

**DOI:** 10.1002/mgg3.825

**Published:** 2019-06-18

**Authors:** Keze Ma, Dong Luo, Tian Tian, Ning Li, Xiaoguang He, Chunbao Rao, Baimao Zhong, Xiaomei Lu

**Affiliations:** ^1^ Department of Neonates Dongguan Children's Hospital Dongguan China; ^2^ Department of Medical and Molecular Genetics Dongguan Institute of Pediatrics Dongguan China; ^3^ Medical Laboratory Dongguan Children's Hospital Dongguan China; ^4^ Department of Pathology The First Affiliated Hospital of Sun Yat‐sen University Guangzhou China

**Keywords:** Chinese, *CRYAB*, infant, muscle performance, myofibrillar myopathy

## Abstract

**Background:**

Due to inconsistencies with reported myofibrillar myopathy (MFM), including autosomal dominant inheritance, late onset and a slowly progressive course, the severe, recessively inherited form of *CRYAB* (alpha‐B crystallin) gene‐related infantile MFM has been suggested. Here, we report an infant in a Chinese family with fatal neonatal‐onset hypertonic MFM with a novel *CRYAB* homozygous variant *(c.3G > A (p.Met1?))*.

**Methods:**

Muscle biopsy indicated that muscle fibers showed a uniformly small diameter, cell atrophy, and visible focal muscle fiber degeneration and necrosis consistent with myogenic myopathy. We performed the whole exome sequencing of pathogenic genes and identified it as MFM.

**Results:**

The proband presented with profound muscle stiffness, progressive respiratory distress and a concurrent abnormal increase in myocardial enzymogram, and the patient died in the 17th month of life. Muscle biopsy and electron microscopy results were consistent with ultramicroscopic myogenic damage and pathological changes. Mutation analysis of the proband identified a novel rare homozygous mutation in the initiation codon of the *CRYAB* gene, which was inherited from currently asymptomatic, heterozygous carrier parents, and his heterozygous biological brother is unaffected.

**Conclusions:**

This article reports one infant with *CRYAB*‐related neonatal onset MFM with a novel homozygous variant in *CRYAB*. To our knowledge, this is the first reported case of infantile alpha‐Bcrystallinopathy in the Chinese population.

**Abbreviations**
ACMG: American College of Medical Genetics
AST: aspartate aminotransferase
ATP: adenosine triphosphatase
CK: creatine kinase
CK‐MB: creatine kinase‐MB
LDH: lactate dehydrogenase
MFMs: Myofibrillar myopathies
MRI: Magnetic resonance imaging
NADH‐TR: NADH‐tetrazolium reductase
NGS: next generation sequencing
ORO: oil red O
PAS: Periodic Acid‐Schiff
sHSP: small heat‐shock protein
TEM: Transmission electron microscopy
α‐HBD: α‐hydroxybutyric dehydrogenase

## 1 BACKGROUND

Myofibrillar myopathies (MFMs) are a genetically heterogeneous group of skeletal and cardiac muscle disorders. A major morphological feature unifying these genetically diverse disorders is that myofibrillar degeneration starts at or close to the Z‐disc of the sarcomere (Nakano, Engel, Waclawik, Emslie‐Smith, & Busis, 1996; Selcen, 2011). MFMs generally affect adults, the clinical features are somewhat more variable, distal muscles are often involved, and cardiomyopathy, peripheral neuropathy and cataracts can be associated features (Selcen, 2011). They can be caused by mutations in different genes with both autosomal dominant and autosomal recessive modes of inheritance (Sacconi et al., 2012). So far, pathogenic mutations associated with MFM phenotype, including atypical MFM‐like cases, have been identified in 17 genes: including six classic genes *DES*, *CRYAB*, *ZASP*, *FLNC*, *BAG3*, *MYOT*, and other genes *FHL1*, *TTN*, *DNAJB6*, *PLEC*, *LMNA*, *ACTA1*, *HSPB8*, *KY*, *PYROXD1*, and *SQSTM* + *TIA1* (digenic) found subsequently. Together they suggested the complicated genetic background of MFM (Jakub Piotr Fichna, Maruszak, & Żekanowski, 2018; Sacconi et al., 2012).

Previous reports have suggested a severe, recessively inherited form of *CRYAB*‐related infantile MFM (Del Bigio et al., 2011; Forrest et al., 2011). The affected child often presents with evident truncal hypertonia, progressive respiratory distress soon after birth, and serum creatine kinase (CK) levels elevated 15–20‐fold above normal; some patients die between 6 weeks and 7 months of age, and some remain alive but ventilator dependent. The *CRYAB* gene (OMIM 123590) is composed of three exons and encodes alpha‐B crystallin, which belongs to the small heat shock protein family.

Here, we report one infant in a Chinese family with *CRYAB*‐associated neonatal‐onset MFM with a novel homozygous *CRYAB* variant and describe the fatal nature of this recessive inheritance (biallelic null mutation). This case may provide new information into the physiological role of alpha‐B crystallin.

## 2 MATERIALS AND METHODS

### 2.1 Ethical Compliance

The study was approved by the Ethics Committee of Dongguan Children's Hospital in agreement with the Declaration of Helsinki. An informed consent was obtained from the parents of the study subject.

### 2.2 Case presentation

This male infant was the second born of healthy, nonconsanguineous Chinese parents. At the age of 2 months, the child began to have difficulty in feeding and increased breathing effort, accompanied by weak hands and feet and mental fatigue. Then, at more than 5 months after birth, the child had a sudden high fever, cough, and progressive respiratory distress. During hospitalization, the preliminary presentation of our patient included severe pneumonia, type II respiratory failure, suspect myocardial involvement, high abdominal pressure, mild malnutrition, and suspect myopathy including myotonic dystrophy, mitochondrial encephalomyopathy, and glycogen storage disease after the initial clinical examination. Breathing was assisted by a ventilator, and a series of treatment were administered including increase nutrients in the myocardium, reduce the cardiac load, strengthen bowel movements and outgassing, improve intestinal function, and low‐dose adrenaline and furosemide. After symptomatic supportive treatment, the child still had abdominal distension, total abdominal muscle tension, and intestinal tympanites, but no significant pneumoperitoneum or ascites was observed on abdominal CT. There was no specific diagnosis of abdominal puncture. Two failed attempts to remove the catheter mainly manifested as respiratory weakness with no obvious cough reflex, and blood gas measurements were indicative of carbon dioxide retention.

Further progression included progressively aggravated muscle stiffness that extended into the trunk, neck and proximal muscles of the limb. Treatments included nutrition for the nerves, anti‐infection medications, and ventilator‐assisted breathing. MRI of the head and spinal cord showed widened extracerebral interspace, a slightly deepened cerebral sulcus, and thickened left temporal and bilateral frontal meninges with significant reinforcement. A muscle MRI showed that bilateral lumbar dorsal muscle signals were abnormal. Muscle biopsy indicated that muscle fibers showed a uniformly small diameter, cell atrophy, and visible focal muscle fiber degeneration and necrosis consistent with myogenic myopathy. Then, symptomatic treatments were conducted, including intravenous piperacillin and sulbactam sodium to prevent infection, airway management, defervescence, and adjusted feeding amounts plus appropriate fluid replacement. During this period, the child occasionally had repeated fevers and lung infections. After electronic bronchofiberscope lavage and anti‐infection treatment based on the patient's symptoms, the child still needed ventilator‐assisted breathing and experienced progressive muscle stiffness, and the creatine kinase (CK) level was continuously elevated (abnormal CK increase, with levels 15–20‐fold above normal). We performed the whole exome sequencing of pathogenic genes and identified it as MFM (*CRYAB*, GeneBank reference sequence: NM_001885.2). The genetic results are essential to diagnose the patient with a form of MFM. There were no cataracts. The proband died at 17 months of age. His parents (father: 34 years old; mother: 31 years old) and biological brother (7 years old) had no clinical manifestations, and there was no family history of myopathy.

### 2.3 Genetic studies

Target capture and sequencing: The genomic DNA of the three patients was extracted from peripheral whole blood samples using the Solpure Blood DNA kit (Magen) and then fragmented with a Q800R Sonicator (Qsonica) to generate 300–500 bp fragments. Custom‐designed NimbleGen SeqCap probes (Roche NimbleGen, Madison, WI) were used for in‐solution hybridization to enrich target sequences. Enriched DNA samples were indexed and sequenced on a NextSeq500 sequencer (Illumina, San Diego, CA) with 100–150 cycles of single‐end reads. The DNA of the proband's brother was Sanger sequenced based on the mutations identified in the proband.

Variant annotation and interpretation: The primary data were in FASTA format after image analysis, and base calling was conducted using the Illumina Pipeline. The data were filtered to generate “clean reads” by removing adapters and low‐quality reads (Q20). Sequencing reads were mapped to the reference human genome version hg19 (2009–02 release, http://genome.ucsc.edu/). Nucleotide changes observed in the aligned reads were called and reviewed using NextGENe software (SoftGenetics, State College, PA). In addition to the detection of deleterious mutations and novel single‐nucleotide variants, a coverage‐based algorithm developed in‐house, eCNVscan, was used to detect large exonic deletions and duplications. Sequence variants were annotated using population and literature databases including 1,000 Genomes, dbSNP, GnomAD, Clinvar, HGMD and OMIM. Some online software (SNP&GO, MutPred, MutationTaster and PolyPhen‐2) was used to analyze the structure of the protein, predict conserved and functional domains and perform multiple sequence alignment. Variant interpretation was performed according to American College of Medical Genetics (ACMG) guidelines (Richards et al., 2015). Possible pathogenicity was predicted according to the online tools MutationTaster and PolyPhen‐2.

## 3 RESULTS

### 3.1 Muscle biopsy

**Figure 1:**
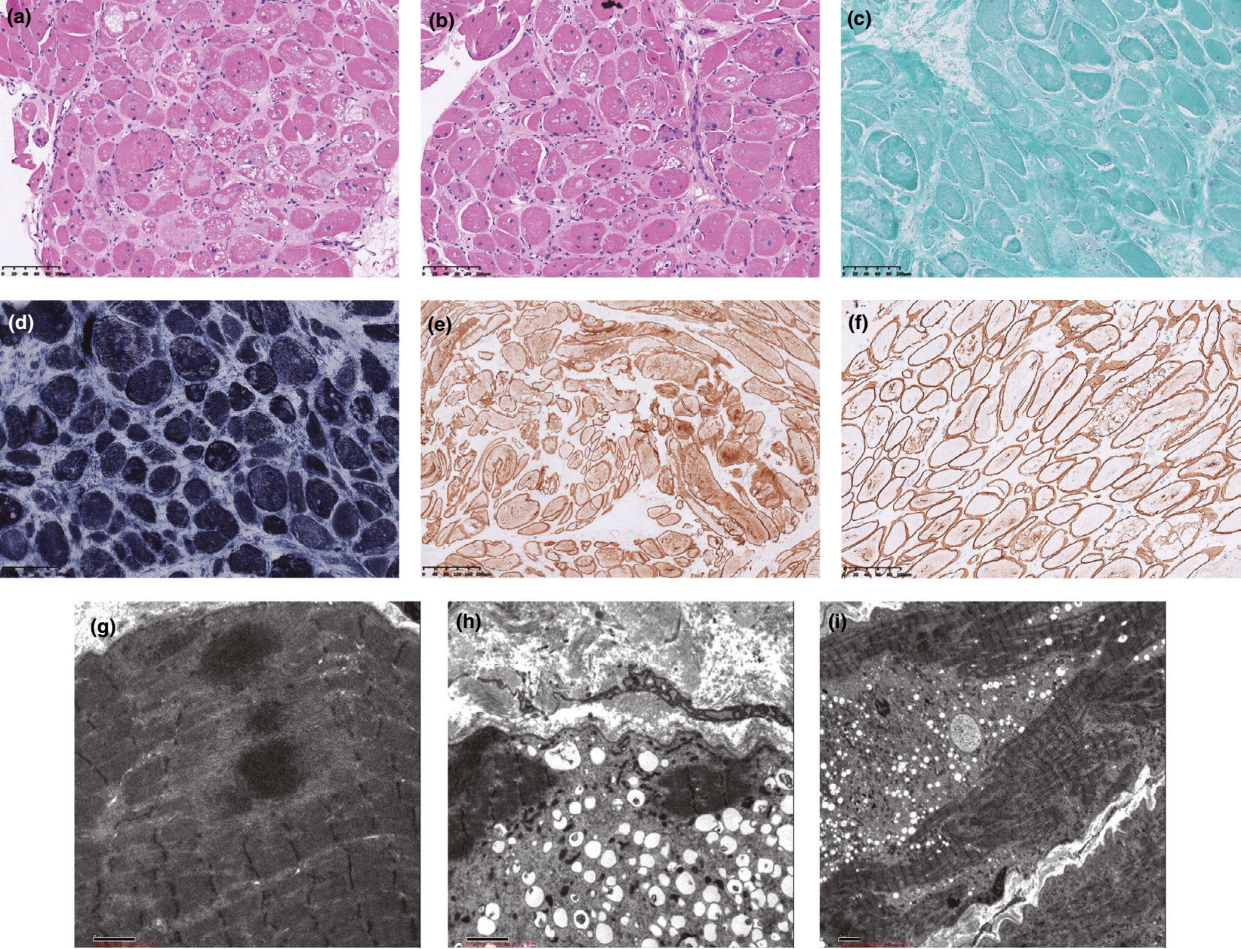
Muscle biopsy findings in the rectus abdominis biopsy (at the age of 6 months). Hematoxylin and eosin staining (a, b) shows visible nuclear ingression, visible focal degeneration, and necrotic muscle fibers. Modified Gomori trichrome (c) staining shows no broken red fiber structures. NADH‐TR (d) staining shows no obvious target/target‐like structures or visible cancellated structure disorder in the muscle fibers. Immunohistochemistry of the rectus abdominis specimen was positive for both dystrophin‐C (e) and dystrophin‐*N* (f). Transmission electron microscopy of the rectus abdominis biopsy (g‐i) showed the loss of myofibrils in some areas of the muscle tissue. The glycogen content in the Z‐line and focal area of some muscle fibers increased significantly. There was visible disorder in the local myofibril arrangement with muscle‐soluble lesion formation and local Z‐band loss. The results suggest ultrastructural pathological changes that are indicative of myogenic damage

Skeletal muscle tissue was examined by staining of paraffin‐embedded and frozen sections. Muscle fibers varied slightly in size, and some muscle fibers show atrophy, a small diameter, and rounding. Nuclear ingressions, focal degeneration, and necrotic muscle fibers were visible (Figure 1a,b). The perimysium and endomysium showed fibrous adipose tissue hyperplasia with no lymphocyte and mononuclear cell infiltration. Modified Gomori trichrome staining of the gastrocnemius and rectus abdominis showed no broken red fiber structures (Figure 1c). NADH‐TR staining showed no obvious target/target‐like structures or visible cancellated structural disorder in the muscle fibers (Figure 1d). ATP, PAS, and ORO staining showed no significant abnormalities. Gastrocnemius and rectus abdominis immunohistochemistry was positive for both dystrophin‐C and dystrophin‐*N* (Figure 1e,f). In this case, the muscle tissue showed a uniformly small diameter, cell atrophy, visible focal degeneration and necrosis, in line with myogenic myopathy.

Transmission electron microscopy (TEM) of the rectus abdominis biopsy further confirmed ultrastructural pathological changes indicative of myogenic damage (Figure 1g‐i). The TEM results showed the loss of myofibrils in some areas of the muscle tissue. The glycogen content in the Z‐line and focal area of some muscle fibers was significantly increased. There was visible local secondary lysosome formation and disorder of local myofibril arrangements with muscle‐soluble lesion formation. The local Z‐band was lost, and the number of mitochondria in some muscle fibers was increased slightly, but no heterotypic mitochondria were observed. There was visible connective tissue hyperplasia between muscle fibers, and little lymphocyte infiltration was observed. However, the TEM of the gastrocnemius biopsy showed only a small amount of muscle fiber atrophy (data not shown). The predilection for truncal muscles may be related to the greater expression of alpha‐B crystallin in type 1 fibers, which are enriched in large postural muscles (Del Bigio et al., 2011; Golenhofen, Perng, Quinlan, & Drenckhahn, 2004). Muscle biopsy and electron microscopy were completed when the patient was 6 months old.

### 3.2 Magnetic resonance imaging (MRI)

MRI of the head and spinal cord showed a widened extracerebral interspace, slightly deepened cerebral sulcus, and thickened left temporal and bilateral frontal meninges with significant reinforcement. Muscle MRI indicated that bilateral lumbar dorsal muscle signals were abnormal.

### 3.3 Cardiac studies

With a diagnosis of probable cardiac involvement, the electrocardiography and echocardiography were performed. There was no abnormality observed by electrocardiogram, and atrial enlargement was observed in the heart during the final stage of the disease in a color Doppler ultrasound. It is worth noting that the CK and CK‐MB values together with other myocardial enzymes (α‐HBD, AST, and LDH) in our proband were abnormally increased and maintained at a high level. The CK‐MB/CK ratio was generally approximately 20%, and cardiac troponin I was initially high.

### 3.4 Genetic studies

**Figure 2:**
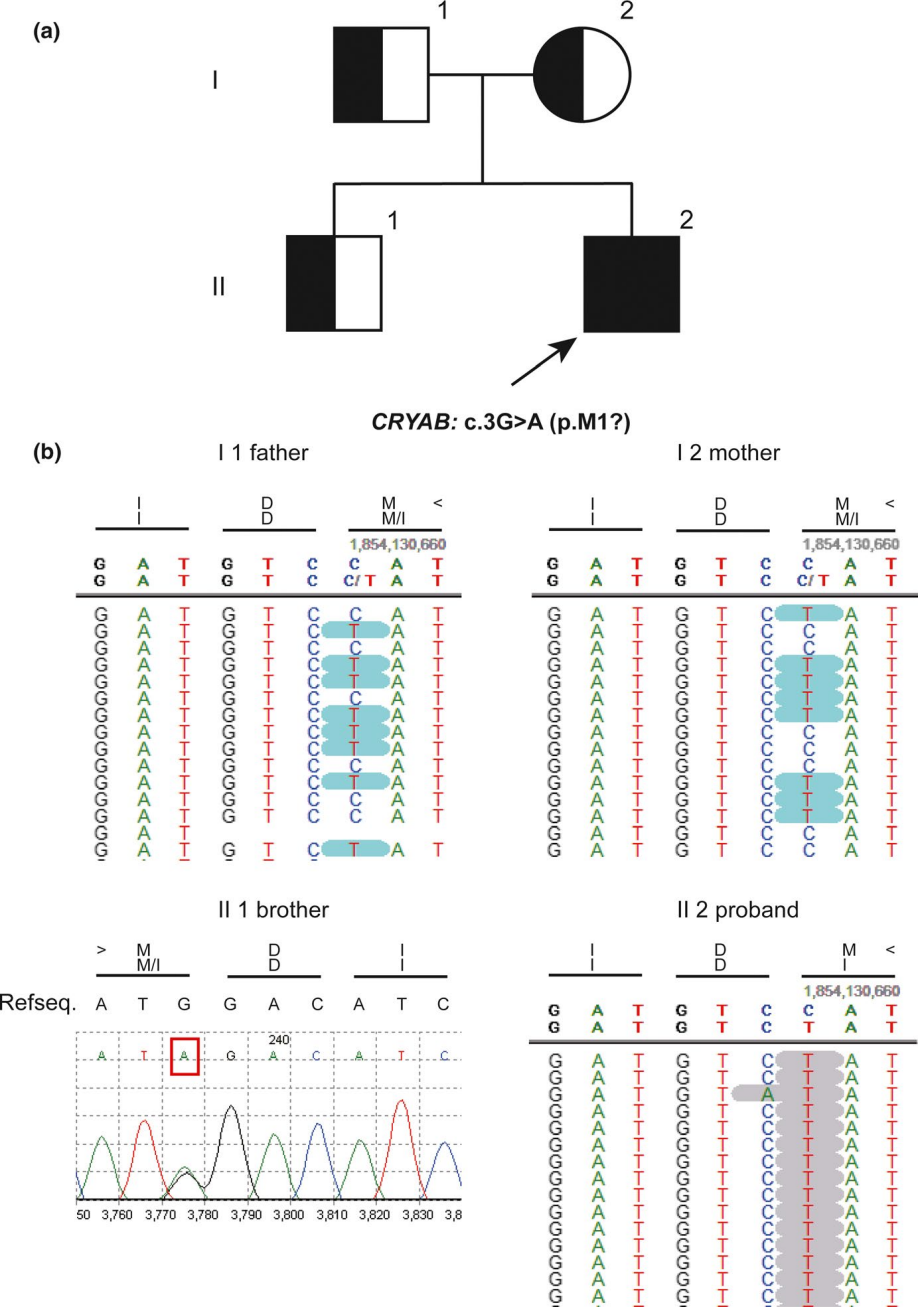
Pedigree of the family (a) and DNA sequencing results (b) of the proband and his family

**Figure 3:**
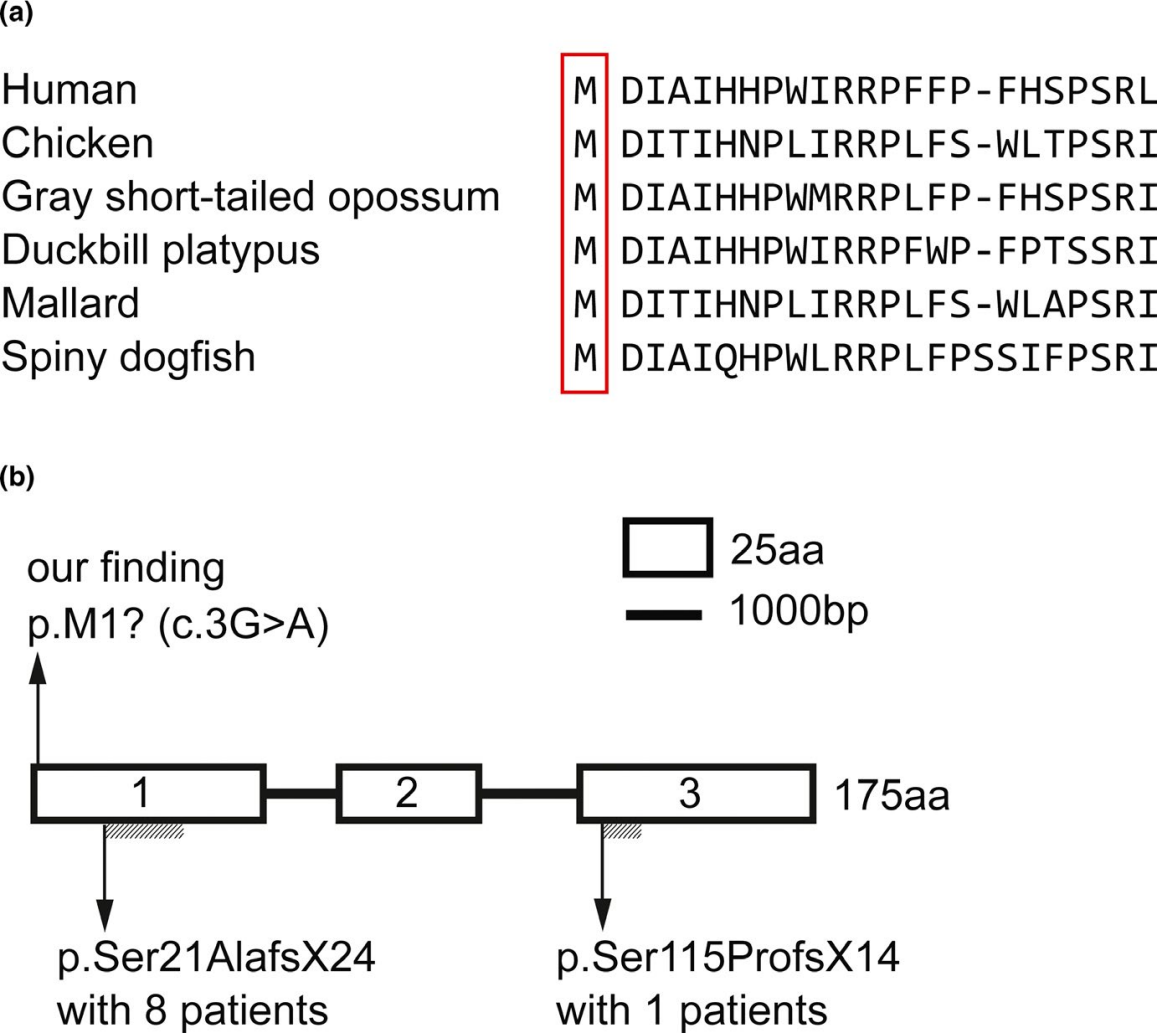
Protein alignment of *CRYAB* in different species, which shows the conservation of the Met1 residue (a). Schematic diagram of alpha‐B crystallin‐related infantile hypertonic MFM (previous finding and our finding); the shaded region represents missense residues (b)

Target gene capture with next generation sequencing (NGS) and gene‐specific Sanger sequencing identified a homozygous *c.3G > A (p.Met1?)* mutation in exon 1 of the *CRYAB* gene (GeneBank reference sequence: NM_001885.2) in the proband, which was inherited from currently asymptomatic, heterozygous carrier parents. A detailed family history (Figure 2a) and DNA sequencing results for the proband and his family (Figure 2b) are provided. The identified mutation is predicted to generate a truncated protein of 108 amino acids or result in the complete absence of the protein. This mutation is predicted to be “probably damaging” by PolyPhen‐2 with a score of 0.999 (sensitivity: 0.14, specificity: 0.99) and “disease causing” by MutationTaster with a score of 1. The methionine at codon 1 is highly conserved in different species. The protein alignment of *CRYAB* in different species (Figure 3a) and schematic diagram of alpha‐B crystallin‐related infantile hypertonic MFM (Figure 3b) are also provided. Variant interpretation was performed according to ACMG guidelines with a very strong evidence of pathogenicity (PVS1), a moderate evidence of pathogenicity (PM2), and two supporting evidences of pathogenicity (PP3 and PP4, detailed in Data S1). Detailed genetic studies including the detailed variant interpretation, the filtering and prioritization procedure and the list of the variants left after final filtering step are provided in the Data S1.

## 4 DISCUSSION AND CONCLUSIONS

To date, *CRYAB*‐related fatal infantile hypertonic MFM (OMIM 613869) has been described in two populations, with the same homozygous mutation *(c.60delC (p.Ser21AlafsX24))* identified in eight Canadian Aboriginal patients (Del Bigio et al., 2011) and another homozygous mutation *(c.343delT (p.Ser115ProfsX14))* identified in a female Caucasian infant (Forrest et al., 2011). In our case, a novel homozygous mutation *(c.3G > A (p.Met1?))* in the *CRYAB* gene was identified, and this rare mutation is predicted to severely affect the synthesis of the *CRYAB* protein by disrupting the translation initiation codon (ATG). The parents and biological brother of our patient are all heterozygous and have no corresponding clinical manifestations. The clinical manifestations of our proband were consistent with the above cases. MFMs usually display a late‐onset, slowly progressive course with variable additional features and are transmitted by autosomal dominant inheritance (Fichna et al., 2017; Reilich et al., 2010; Selcen & Engel, 2003), but in this disease, a more severe form and autosomal recessive inheritance in a similar genetic background were suggested. Consistent with previous reports (Del Bigio et al., 2011; Forrest et al., 2011), our finding suggests the fatal nature of this recessive inheritance (biallelic null mutation).

**Figure 4:**
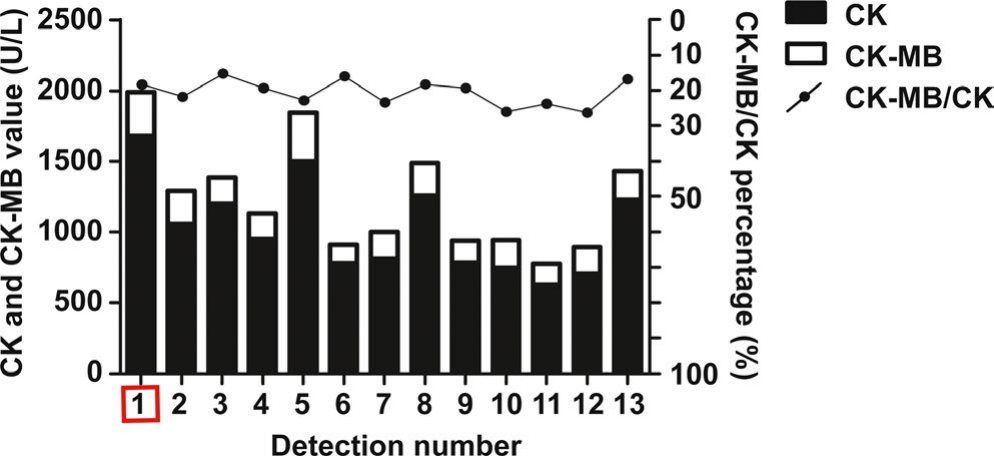
The measured CK and CK‐MB values of our proband. The CK and CK‐MB values, together with other myocardial enzymes, were abnormally increased and maintained at a high level. We selected 13 test results in chronological order, including the initial measurement (in the box), to represent the general condition. The CK‐MB/CK ratio was generally approximately 20%. Cardiac troponin I was initially high. There was no abnormality in the electrocardiogram, and atrial enlargement was observed in the final stage of the disease through color Doppler ultrasound of the heart, but no serious heart dysfunction was observed

There was no evidence of additional features, including cardiomyopathy, peripheral neuropathy, or cataracts, which have been described in previous reports of infantile alpha‐Bcrystallinopathy (Del Bigio et al., 2011; Forrest et al., 2011). In our patient, there was no peripheral neuropathy or cataracts. However, we noticed that the CK and CK‐MB values together with other myocardial enzymes (α‐HBD, AST, and LDH) in our proband were abnormally increased and maintained at a high level. The CK‐MB/CK ratio was generally approximately 20% (6%–25% suggest creatine kinase with a myocardial origin), and cardiac troponin I was initially high (Figure 4); however, there was no abnormality observed by electrocardiogram, and atrial enlargement was observed in the heart during the final stage of the disease in a color Doppler ultrasound. Although no serious heart dysfunction was observed in our patient, the possibility of myocardial damage was suggested. Cardiomyopathy can present as an end‐stage progression of certain systemic myopathies in children or adults, or cardiac symptoms can sometimes precede muscle weakness as the initial presentation (Katzberg, Karamchandani, So, Vogel, & Wang, 2010). In a report of desminopathy, there was severe infantile‐onset cardiomyopathy associated with a homozygous deletion in desmin (Piñol‐Ripoll et al., 2009), but this has not been described in alpha‐Bcrystallinopathy in infancy. Additionally, the heart of alpha‐B crystallin knockout mice (Brady et al., 2001) is unaffected, but the relevant mechanism is still unclear. In mammals, cytoskeletal network maintenance of cardiomyocytes (rescue pathway) may be more complex and diverse than that of skeletal muscle cells.

Here, the role of the *CRYAB* protein in muscle performance needs to be discussed in‐depth again. alpha‐B crystallin belongs to the small heat‐shock protein (sHSP) family (Haley, Horwitz, & Stewart, 1998), and its primary role is to bind unfolded and denatured proteins to suppress nonspecific aggregation. alpha‐B crystallin is found in lens and nonlenticular tissues, with the highest levels in cardiac and skeletal muscle. In these tissues, alpha‐B crystallin is immunolocalized to the Z‐disk, and its expression is enhanced after stress and exercise (Djabali, de Néchaud, Landon, & Portier, 1997; Neufer, Ordway, & Williams, 1998). Recently, research on the *CRYAB* protein in *Drosophila* provided further evidence that this protein is required for myofibril integrity and muscle performance (Wojtowicz et al., 2015). *CRYAB* displays muscle‐ and heart‐specific expression and accumulates at Z‐bands. As a chaperone of the type III IF protein desmin, alpha‐B crystallin helps to maintain cytoskeletal integrity in skeletal and cardiac muscle cells (Golenhofen et al., 1999; Wojtowicz et al., 2015). The clinical characterization of our patient (disease development in the trunk and possible myocardial involvement) and electron microscopy results (for instance, local Z‐band loss) support this interpretation. However, in the mammalian heart, the condition may be more complex. Desmin IFs are mainly associated with Z‐bands and play an important role in the spatial organization of the contractile apparatus through interactions with other sarcomeric proteins to construct an integral cytoskeletal network (Goldfarb & Dalakas, 2009). Therefore, the *CRYAB* protein was thought to help maintain myofibrillar architecture by interacting with sarcomeric components and, in particular, with Z‐band‐associated proteins. Furthermore, both muscle‐specific knockdown of *CRYAB* in *Drosophila* (Wojtowicz et al., 2015) and alpha‐B crystallin knockout mice (Brady et al., 2001) provide further evidence and share the typical MFM phenotype. In addition, Del Bigio et al. (2011) provide a possible explanation for the reduced muscle elasticity, that the *CRYAB* interaction with titin is disrupted (Del Bigio et al., 2011).

The *c.3G > A* mutation identified in this study affects the initiation codon of the *CRYAB* gene and is likely, therefore, to affect translation initiation of *CRYAB* mRNA. Methionine at codon 1 is highly conserved in different species. When there is a mutation in the initiation codon, it is possible for an alternative AUG within the transcript to be used as an aberrant translation initiation site (Uzak, Tokgoz, Dundar, & Tekin, 2013). There is an ATG triplet in exon 2,201 bp downstream of the authentic initiation codon. Even if that ATG was used as an initiation codon, the resulting truncated protein would lack 67 amino acids. However, some experiments using different *CRYAB* mutants have demonstrated that the C‐terminal extension (residues 106–175) is important for alpha‐B crystallin self‐interaction (Fu & Liang, 2002) and for oligomerization (Hayes, Devlin, & Quinlan, 2008). Thus, based on the above‐mentioned and limited variability observed in our patients, we suggest that the identified mutation has a profound impact on the protein, which is most likely completely absent, or the condition reflects a quantitative effect resulting from homozygosity for a truncating *CRYAB* mutation (Forrest et al., 2011) and is sufficient to produce such a severe phenotype. The relevant mechanism still needs further research, especially in mammals, to determine the unidentified discrepancy responsible for the high expression of alpha‐B crystallin in both cardiomyocytes and skeletal muscle cells. In addition, it is worth noting that, the variant was already twice reported in Clinvar (Clinvar id 44236) and has a number in dbSNP database (rs397516686). However, there are no definitive case reports providing strong evidence of pathogenicity (Conflicting interpretations of pathogenicity). Our study provides clear evidence of the pathogenesis of the mutation and expands the clinical variation data for the disease. At the same time, we also noticed that there is a homozygote reported in ExAC database, however its total allele frequency is extremely low (7.562e‐05). For this discrepancy, we speculate that there is a possible involvement of complex genetic background, it is hard to pinpoint, or unlikely detected by WES.

In conclusion, this article reports one infant with *CRYAB*‐related neonatal onset MFM with a novel homozygous variant in *CRYAB*. This is the first reported case of infantile alpha‐Bcrystallinopathy in the Chinese population to date, and our work has important implications for further elucidating the pathogenic mechanism of this MFM that is fatal in infancy. In addition, this study may aid in diagnosis, carrier detection, and genetic counseling.

## CONFLICT OF INTEREST

We declare that we have no conflicts of interest.

## AUTHOR CONTRIBUTIONS

KZM, DL, BMZ, and XML conceived and designed the work; KZM, DL, XGH, CBR, XML, TT, BMZ, and NL performed the experiments, KZM, DL, XGH, CBR, BMZ, and NL analyzed the data. KZM, DL, BMZ, and XML wrote the manuscript. All authors read and approved the final manuscript.

## ETHICS APPROVAL AND CONSENT TO PARTICIPATE

The study was approved by the Ethics Committee of Dongguan Children's Hospital in agreement with the Declaration of Helsinki. An informed consent was obtained from the parents of the study subject.

## CONSENT FOR PUBLICATION

All members of the family signed a written consent for publishing all the data.

## Data Availability

All relevant data are included in the manuscript. The datasets used and/or analysed during the current study are available from the corresponding author upon request.
