# Supplementary material for: A novel homozygous initiation codon variant associated with infantile alpha‐Bcrystallinopathy in a Chinese family
Source: Mol Genet Genomic Med. 2019 Jun 18;7(8):e825. doi: 10.1002/mgg3.825 (PMC6687638; doi:10.1002/mgg3.825)
Supplement: Supplementary file 1 [file MGG3-7-e825-s001.docx]

**Supplementary material**

Detailed genetic studies

1. Target capture and sequencing

Genomic DNA from the family was extracted from peripheral whole blood samples using the Solpure Blood DNA kit (Magen) according to the manufacturer’s instructions. The genomic DNA of the three patients was then fragmented with a Q800R Sonicator (Qsonica) to generate 300 - 500 bp fragments. The paired-end libraries were prepared according to the Illumina library preparation protocol. Custom-designed NimbleGen SeqCap probes (Roche NimbleGen, Madison, WI) were used for in-solution hybridization to enrich target sequences. Enriched DNA samples were indexed and sequenced on a NextSeq500 sequencer (Illumina, San Diego, CA) with 100–150 cycles of single-end reads, according to the manufacturer’s protocols. The DNA of the proband’s brother was Sanger sequenced based on the mutations identified in the proband.

2. Variant annotation and interpretation

The primary data was in FASTA format after image analysis, and base calling was conducted using the Illumina Pipeline. The data were filtered to generate ‘clean reads’ by removing adapters and low-quality reads (Q20). Sequencing reads were mapped to the reference human genome version hg19 (2009-02 release, http://genome.ucsc.edu/). Nucleotide changes observed in the aligned reads were called and reviewed using NextGENe software (SoftGenetics, State College, PA). In addition to the detection of deleterious mutations and novel single-nucleotide variants, a coverage-based algorithm developed in-house, eCNVscan, was used to detect large exonic deletions and duplications. The normalized coverage depth of each exon of a test sample was compared with the mean coverage of the same exon in the reference file to detect copy number variants (CNVs). The filtering and prioritization procedure and the list of the variants left after final filtering step in Figure 2 and Table 3 below.

Sequence variants were annotated using population and literature databases including 1000 Genomes (http://www.1000genomes.org/), dbSNP (http://www.ncbi.nlm.nih.gov/), GnomAD (http://gnomad.broadinstitute.org/), Clinvar (https://www.st-va.ncbi.nlm.nih.gov/clinvar/), HGMD (http://www.hgmd.cf.ac.uk/ac/index.php) and OMIM (https://www.omim.org/). Some online software (SNP&GO, MutPred, MutationTaster and PolyPhen-2) was used to analyze the structure of the protein, predict conserved and functional domains and perform multiple sequence alignment. Variant interpretation was performed according to American College of Medical Genetics (ACMG) guidelines. Possible pathogenicity was predicted according to the online tools MutationTaster and PolyPhen-2.

1. **Allele(s) Basic information**

NM_001885.2(CRYAB):c.3G>A (p.Met1Ile) (ClinVar)

| Transcript ID | NM_001885/ ENST00000533475 |
| --- | --- |
| Allele ID | 53403 |
| UniProt peptide | P02511 |
| Variant type | single nucleotide variant |
| Cytogenetic location | 11q23.1 |
| Genomic location | Chr11: 111911722 (on Assembly GRCh38)  Chr11: 111782446 (on Assembly GRCh37) |
| Protein change | Met1Ile |
| Other names | p.Met1? |

1. **Pathogenic: PVS1+ PM2 + PP3 + PP4**

**Table 1.** The detailed criteria (ACMG Standards and Guidelines) for our classification.

| *Very strong evidence of pathogenicity*  **PVS1** Null variant (nonsense, frameshift, canonical +/−1 or 2 splice sites, initiation codon, single or multi-exon deletion) in a gene where loss of function (LOF) is a known mechanism of disease. |
| --- |
| *Moderate evidence of pathogenicity*  **PM2** Absent from controls (or at extremely low frequency if recessive) in Exome Sequencing Project, 1000 Genomes or ExAC. |
| *Supporting evidence of pathogenicity*  **PP3** Multiple lines of computational evidence support a deleterious effect on the gene or gene product (conservation, evolutionary, splicing impact, etc).  **PP4** Patient’s phenotype or family history is highly specific for a disease with a single genetic etiology. |

1. **Interpretation**

**PVS1**: Null variant (initiation codon) in CRYAB gene where loss of function (LOF) is a known mechanism of disease. (Detailed in manuscript and Figure 1C below)

**PM2**: Absent from controls (or at extremely low frequency if recessive) in Exome Sequencing Project, 1000 Genomes or ExAC. (Table 2)

**Table 2.** The population allele frequencies of the variant.

| Population | Allele: frequency | |
| --- | --- | --- |
| ALL | C: 0.9999306593 | T: 6.93407e-05 |
| African/African American | C: 1.000 | T: 0.000 |
| Latino | C: 1.000 | T: 0.000 |
| Ashkenazi Jewish | C: 1.000 | T: 0.000 |
| East Asian | C: 0.999 | T: 0.001 |
| Finnish | C: 1.000 | T: 0.000 |
| Non-Finnish European | C: 1.000 | T: 0.000 |
| Other | C: 1.000 | T: 0.000 |
| ExAc_Aggregated_Populations | C: 0.99995 | T: 4.97611e-05 |

*Highest minor frequency observed in any population from 1000 Genomes Phase 3, ESP and ExAC: < 0.01

**PP3**: Predicted to be “probably damaging” by PolyPhen-2 with a score of 0.999 (sensitivity: 0.14, specificity: 0.99) (<http://genetics.bwh.harvard.edu/pph2>) and “disease causing” by MutationTaster with a score of 1 (<http://www.mutationtaster.org>). The methionine at codon 1 is highly conserved in different species. (Figure 1A below)

**PP4**: Pedigrees of the family members are shown documenting the segregation of alleles. (Figure 1B below)


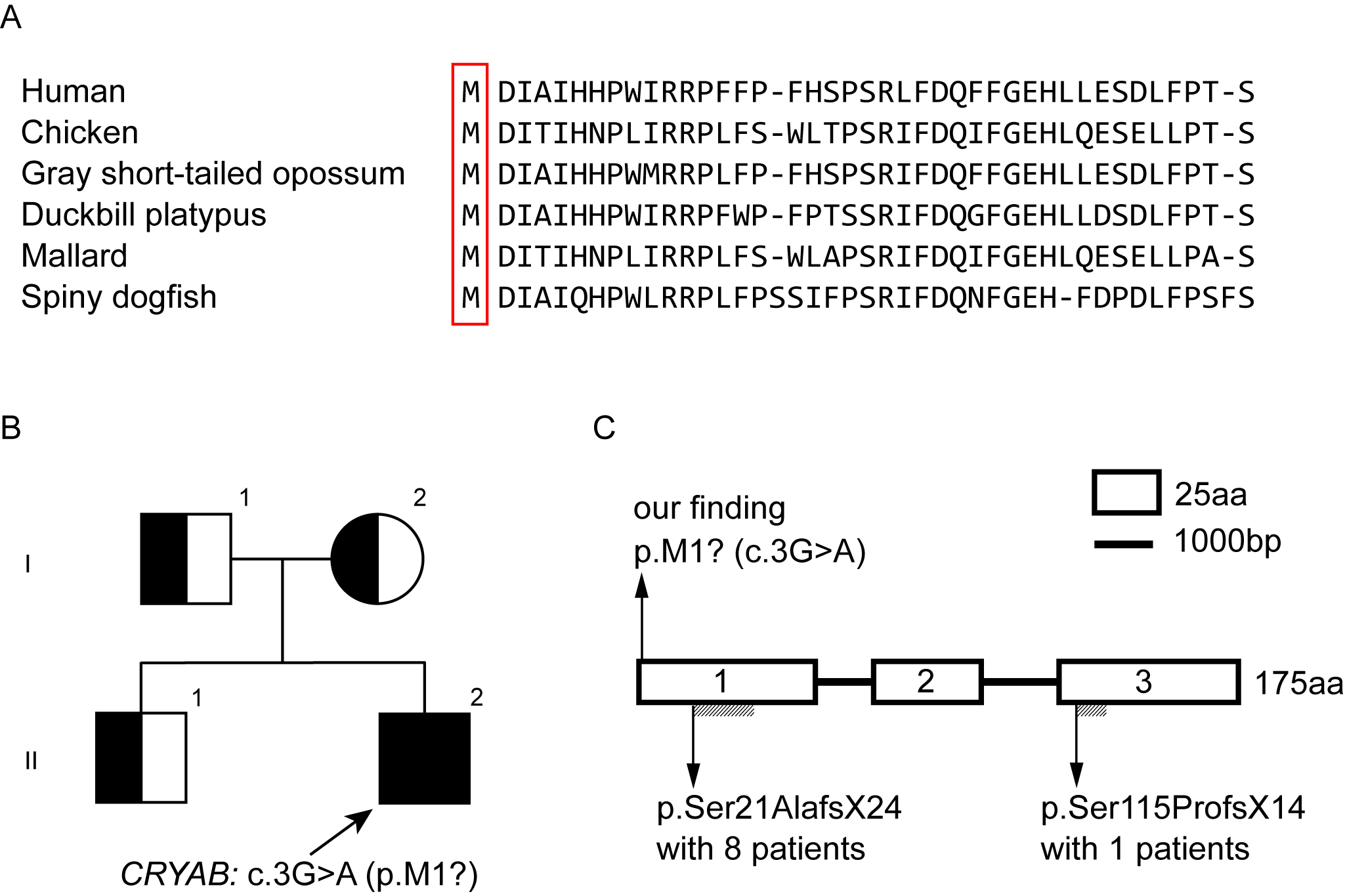


**Figure 1.** Protein alignment of CRYAB in different species, which shows the conservation of the Met1 residue (A). Pedigrees of family members are shown documenting the segregation of alleles (B). Schematic diagram of αB-crystallin-related infantile hypertonic MFM (previous finding and our finding); the shaded region represents missense residues (C).


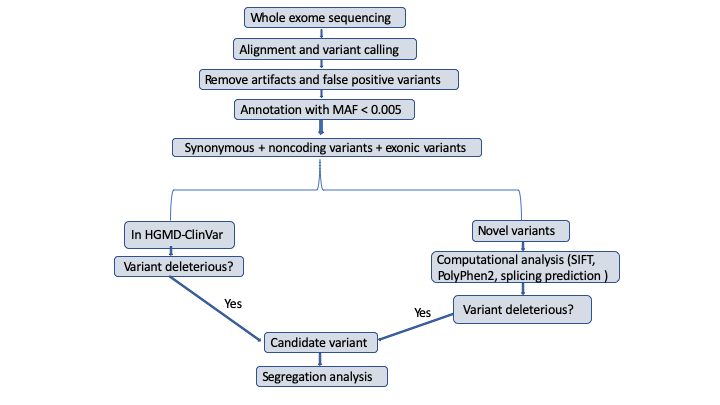


**Figure 2.** The filtering and prioritization procedure.

**Table 3.** The list of the variants left after final filtering step.

| **Index** | **Chr** | **Chr Position** | **Filter Chr:Position** | **Gene** | **ACMGG** | **Source** | **Function** | **Nucleotide Change** | **Amino Acid Change** | **Splice** | **Mutant Allele Frequency** | **Father MAF** | **Mother MAF** |
| --- | --- | --- | --- | --- | --- | --- | --- | --- | --- | --- | --- | --- | --- |
| 696 | 1 | 93301815 | 1:93301815 | RPL5 | 3 | D | Synonymous | c.393T>C | p.N131N |  | 11.36 | 0 | 0 |
| 1059 | 1 | 155764972 | 1:155764972 | GON4L | 3 | D | Noncoding | c.1646-30A>T |  |  | 15.22 | 0 | 0 |
| 1409 | 1 | 203472041 | 1:203472041 | OPTC | 2 | D | Noncoding | c.733-1G>T | Splice | 1 | 8.57 | 0 | 0 |
| 2331 | 2 | 109365628 | 2:109365628 | RANBP2 | 3 | D | Noncoding | c.1273+43T>G |  |  | 16.67 | 0 | 0 |
| 3723 | 3 | 55508494 | 3:55508494 | WNT5A | 3 | D | Missense | c.555C>G | p.N185K |  | 7.14 | 0 | 0 |
| 4213 | 3 | 195595248 | 3:195595248 | TNK2 | 4 | MC | Missense | c.2110G>A | p.A704T |  | 50 | 0 | 54.02 |
| 4214 | 3 | 195606096 | 3:195606096 | TNK2 | 3 | PC | Noncoding | c.1077-50C>T |  |  | 51.43 | 39.36 | 0 |
| 4275 | 4 | 3475227 | 4:3475227 | DOK7 | 3 | D | Synonymous | c.195G>T | p.L65L |  | 7.41 | 0 | 0 |
| 5044 | 5 | 6616791 | 5:6616791 | NSUN2 | 3 | D | Noncoding | c.1021+49T>A |  |  | 7.14 | 0 | 0 |
| 5891 | 6 | 31084620 | 6:31084620 | CDSN | 3 | D | In-Frame | c.770_772delTGG | p.V257del |  | 7.65 | 0 | 0 |
| 6569 | 6 | 129468231 | 6:129468231 | LAMA2 | 3 | PC | Noncoding | c.909+38C>T |  |  | 52.46 | 54.43 | 0 |
| 6575 | 6 | 129691111 | 6:129691111 | LAMA2 | 3 | MC | Synonymous | c.4935C>T | p.T1645T |  | 63.95 | 0 | 40.46 |
| 8552 | 9 | 32986037 | 9:32986037 | APTX | 3 | D | Noncoding | c.484-9T>C |  |  | 8.16 | 0 | 0 |
| 10926 | 11 | 111782446 | 11:111782446 | CRYAB | 3 | R | Missense | c.3G>A | p.M1I | 3 | 100 | 44.10 | 46.83 |
| 11495 | 12 | 48369784 | 12:48369784 | COL2A1 | 3 | D | Missense | c.3559C>T | p.P1187S |  | 55.07 | 0 | 0 |
| 12290 | 13 | 26586788 | 13:26586788 | ATP8A2 | 3 | D | Noncoding | c.3469+28A>T |  |  | 8.7 | 0 | 0 |
| 13033 | 15 | 28459051 | 15:28459051 | HERC2 | 3 | D | Missense | c.6623C>A | p.A2208D |  | 7.69 | 0 | 0 |
| 13263 | 15 | 44890432 | 15:44890432 | SPG11 | 3 | D | Noncoding | c.4001+31T>G |  |  | 7.32 | 0 | 0 |
| 14992 | 17 | 18061149 | 17:18061149 | MYO15A | 3 | D | In-Frame | c.8903_8911delCAGCCGCCG | p.A2968_A2970del |  | 29.63 | 0 | 0 |
| 15001 | 17 | 18061158 | 17:18061158 | MYO15A | 3 | D | In-Frame | c.8912_8932delTGGCCGCTGCTGTGGCCTCTG | p.V2971_S2977del |  | 36.36 | 0 | 0 |
| 18138 | 22 | 50906927 | 22:50906927 | SBF1 | 3 | D | Noncoding | c.56-37C>G |  |  | 7.55 | 0 | 0 |
| 18179 | X | 8538712 | X:8538712 | KAL1 | 4 | U | Missense | c.890G>A | p.R297Q |  | 100 | 50.00 | 0 |
| 18189 | X | 11136641 | X:11136641 | HCCS | 3 | U | Missense | c.422A>G | p.D141G |  | 97.67 | 60.81 | 0 |
| 18321 | X | 49104054 | X:49104054 | CCDC22 | 3 | D | Noncoding | c.972+35C>A |  |  | 7.27 | 0 | 0 |
| 18378 | X | 69715285 | X:69715285 | DLG3 | 3 | U | Missense | c.1801T>C | p.Y601H |  | 100 | 49.76 | 0 |
